# Thematic roles: Core knowledge or linguistic construct?

**DOI:** 10.3758/s13423-019-01634-5

**Published:** 2019-07-09

**Authors:** Lilia Rissman, Asifa Majid

**Affiliations:** 1 Center for Language Studies, Radboud University, Nijmegen, The Netherlands; 2 Max Planck Institute for Psycholinguistics, Nijmegen, The Netherlands; 3 Department of Psychology, University of York, York, UK

**Keywords:** Semantics, Concepts and categories, Psycholinguistics, Cognitive development

## Abstract

The status of thematic roles such as Agent and Patient in cognitive science is highly controversial: To some they are universal components of core knowledge, to others they are scholarly fictions without psychological reality. We address this debate by posing two critical questions: to what extent do humans represent events in terms of abstract role categories, and to what extent are these categories shaped by universal cognitive biases? We review a range of literature that contributes answers to these questions: psycholinguistic and event cognition experiments with adults, children, and infants; typological studies grounded in cross-linguistic data; and studies of emerging sign languages. We pose these questions for a variety of roles and find that the answers depend on the role. For Agents and Patients, there is strong evidence for abstract role categories and a universal bias to distinguish the two roles. For Goals and Recipients, we find clear evidence for abstraction but mixed evidence as to whether there is a bias to encode Goals and Recipients as part of one or two distinct categories. Finally, we discuss the Instrumental role and do not find clear evidence for either abstraction or universal biases to structure instrumental categories.

## Introduction

Thematic roles such as Agent, Patient, and Goal have a long-standing presence in theories of linguistics and cognitive science. By way of illustration, the verb *eat* encodes a relation between someone who eats and something that gets eaten: these participants have been given the role labels Agent and Patient, respectively. Thematic roles are routinely invoked in studies of the syntax~semantics interface, language production and comprehension, and child language learning. They are argued to be part of innate, core knowledge (Carey, 2009; Strickland, 2016), to be cross-culturally universal (Fillmore, 1968), and to have played a pivotal role in language evolution (Calvin & Bickerton, 2000). Despite this prevalence, discussion of the problematic nature of thematic roles also has a long-standing tradition. Dowty (1991) states “there is perhaps no concept in modern syntactic and semantic theory which is so often involved in so wide a range of contexts, but on which there is so little agreement as to its nature and definition, as THEMATIC ROLE” (p. 547). Nearly 20 years later, Newmeyer (2010) evinces a similar sentiment: “there is no construct as murky in ANY subdivision of linguistic theory as that of ‘thematic role’. Literally dozens have been proposed over the years, and nothing approaching a consensus has been achieved in terms of delineating the set that are needed for natural language semantics” (p. 689).

The literature documenting the murkiness of thematic roles is in fact so vast that we take it as given that individual roles cannot be defined in terms of necessary and sufficient conditions, at least, not in a way that has explanatory and predictive power in cognitive science (Cruse, 1973; DeLancey, 1991; Dowty, 1991; Levin & Rappaport-Hovav, 2005; Schlesinger, 1989). Taking the example of the Instrument role, a wide range of participants have Instrument-like properties (Koenig, Mauner, Bienvenue, & Conklin, 2008; Nilsen, 1973; Rissman & Rawlins, 2017; Schlesinger, 1995):^1^

1. a. Janine ate the custard with a spoon.
  b. Wanda accidentally drew on her shirt with a marker.
  c. Renée applied the lipstick with her fingertips.
  d. Carlos carried the milk in a bucket.
  e. Anita went to Amsterdam by train.
  f. The cue ball hit the red ball which sunk the eight ball.
  g. Tyrell used the steamy room to practice yoga.
  h. The bomb blast destroyed the abandoned factory.
  i. The program completed the algorithm in five seconds.

We defy readers to propose a single generalization that captures what all these roles have in common (without being so broad as to include, say, all event participants).

Despite the difficulty of defining thematic roles, 50 years after the publication of Fillmore’s seminal 1968 paper “The case for case,” thematic roles are still common in scientific parlance and theory because of the possibility that they are fundamental to how human minds develop, how we represent the world, and how we express these representations in language. And although thematic roles are definitionally opaque, this makes them no different from other human concepts (Malt, Gennari, Imai, Ameel, Saji, & Majid, 2015). In one view of the relationship between language and thought, language develops “by linking linguistic forms to universal, pre-existing representations of sound and meaning” (Hespos & Spelke, 2004: p. 453). Applying this nativist view to the thematic role Agent, for example, it may be that Agent is a universal conceptual category present early in life that remains largely unchanged throughout development. It is well-established that the syntax~semantics interface is variable across languages (Bowerman & Brown, 2008; Croft, 2012; Rappaport-Hovav & Levin, 1998). The semantic structures encoding thematic roles, and the conceptual structures interfacing with these semantic structures, may vary relatively little, however.

Such a state of affairs would have wide-ranging implications for theories of linguistic representation, language development, and event representation. Many generative theories of syntax and semantics encode agency via the primitive predicate *Ag(e)* in the truth-conditions of a sentence (Kratzer, 1996; Lohndal, 2014; Parsons, 1990; Schein, 2002; cf. Davidson, 1967). The truth-conditions of *Jim drank champagne*, for example, would contain the proposition *Ag(e) = Jim*, where *e* is a linguistic representation of an event. Across languages, diverse linguistic forms contribute agency to the meaning of a sentence (e.g., nominative case in one language, ergative case in another). Nonetheless, if the nativist view of agency is correct, then the semantic content of *Ag(e)* is the same in every language. For children, if the nativist view of agency is correct, language learning would involve discovering which linguistic form(s) encode the role Agent, rather than constructing this category based on conceptual, linguistic, and cultural experience; and for adults perceiving events in the world, recognition and conceptualization of agents would proceed in the same way in every cultural context.

A variety of evidence suggests that for the Agent role, the nativist view may in fact be correct. The strength of this evidence does not, however, imply that all thematic roles are innate universals. For the Recipient, Goal, and Instrument roles, evidence for the nativist view is more tenuous: the current literature on these roles is more consistent with theories in which conceptual and linguistic categories are constructed, and influence each other over the course of development. On closer examination, even for the Agent and Patient roles, the nativist view is not fully supported because we lack the detailed behavioral evidence required to fully understand the structure of these categories. Previous studies have focused more on elucidating the relationship between roles, rather than uncovering the structure of individual roles. Without a detailed understanding of how role categories are structured, we cannot address whether roles are truly cross-linguistically universal.

We support this argument in two steps. First, we ask whether thematic roles correspond to psychologically real categories. The human ability to create categories is ubiquitous, enabling us to interpret and learn from new experiences. As such, when humans encode events and the relationships between event participants, we expect *a priori* that event participants are represented in terms of categories. The existence of lexically-specific roles (e.g., one who eats, thing that gets eaten) is fairly uncontroversial. However, one can also ask whether there are abstract categories that range over lexically-specific roles, for example, a category that includes not only the thing that gets eaten, but also the thing that gets cleaned, broken, and so forth. Such abstractions would support novel verb learning, efficient sentence processing and rapid event apprehension, among other cognitive abilities.

In the psychological study of concepts, categories are often probed through studying the meanings of words – researchers have asked, for example, about the structure of the category *bird*. Thematic roles, however, often do not map cleanly onto words. For example, although *agent, patient*, and *instrument* are English words, there is only a loose connection between the commonly known meanings of these words and the event categories that researchers have labeled Agent, Patient, and Instrument. Instead, role categories have been thought to reveal themselves through a range of linguistic structures, both lexical (e.g., case markers, adpositions) and syntactic (e.g., grammatical functions such as Subject and Object). In addition, lexical markers of thematic roles are often closed-class and have wide semantic extension, seemingly encompassing multiple meanings and multiple thematic roles. The English preposition *with*, for example, marks instruments, as in (1a), but it also ranges over other roles, as in *I want the room withtwo beds, Marnie delivered the talk withconfidence,* and *I walked to the store withmy brother*.

Given that event participant categories are not as self-evident as categories provided by nouns and verbs, we review studies demonstrating the psychological reality of abstract roles. It is clear that a variety of event- and verb-specific knowledge is activated during sentence comprehension (Bicknell, Elman, Hare, McRae, & Kutas, 2010; Ferretti, McRae, & Hatherell, 2001; McRae, Ferretti, & Amyote, 1997; McRae, Hare, Elman, & Ferretti, 2005; Metusalem, Kutas, Urbach, Hare, McRae, & Elman, 2012). For example, the sentence *the journalist checked the spelling* is processed more quickly than *the mechanic checked the spelling* (Bicknell et al., 2010). Given this detailed event- and verb-specific knowledge, in combination with the difficulty of defining and identifying thematic roles, the question arises as to whether abstract participant categories have a place in a theory of cognition. They may be useful labels in research but lack psychological reality, akin to the “comparative concepts” in Haspelmath (2010). As described by Haspelmath, comparative concepts are “specifically designed for the purpose of comparison [across languages]” (p. 664). That is, they are part of the linguist’s toolkit rather than an aspect of human cognition to be studied in and of itself.

Additionally, some theories of the syntax~semantics interface do not employ thematic roles in their machinery. The analysis in Croft (2012), for example, involves direct mappings between *specific* event structures and syntactic positions (e.g., Subject and Object), rather than incorporating abstract roles. In addition, Rissman and Rawlins (2017) analyze the meanings of the English instrumental markers *with* and *use* only in terms of the roles Agent and Patient, without appealing to the role Instrument. Theories of language and cognition that do without thematic roles, or employ a more restricted set of roles, may be simpler and therefore preferred. It is crucial, therefore, to put forward evidence that proposed thematic roles have psychological reality. Such evidence is found in experiments on event and sentence processing by adults and children, which we review below.

The second step of our argument is to address whether abstract roles in specific languages and cultures are shaped by universal biases in conceptual and semantic structure. Fillmore (1968), for example, proposed that the repertoire of role categories is the same across languages. This hypothesis is not possible to test directly (at least not yet; see fifth section), because experimental studies demonstrating abstract roles have not been conducted in every language. Nonetheless, we review four types of evidence that bear on this question indirectly: linguistic typology, studies of conceptual representation in infancy, sign language emergence, and studies of child language development. As core knowledge is a source of universal cognitive biases, this evidence can be used to support the claim that thematic roles are part of core knowledge.

Linguistic typology involves comparing large sets of diverse languages. Through patterns of colexification across languages, this research sheds light on the extent to which there are universal biases to categorize event roles: when a single adposition or case marker is frequently used to encode two distinct meanings, this indicates that the two meanings are semantically related; when two meanings are frequently marked in different ways linguistically, this indicates that the meanings are semantically more distant. This colexification evidence thus shows common patterns in what types of roles are often distinguished and what roles are often conflated. As the very nature of categories is to distinguish in-group from out-group members, these patterns suggest what notions are regularly distinguished in diverse languages and consequently give clues as to what the categories in individual languages are likely to be.

Even if the same psycholinguistic tasks were conducted on all the languages of the world, and the categories turned out to be the same, we would still not know *why* languages/cultures are the same. Common patterns cross-linguistically could reflect cognitive biases, social structure, historical and environmental influences, or some combination of these factors. We therefore review evidence from infancy, emerging sign language, and child development: developmental data are critical to reveal notions distinguished early in life before language has been fully acquired, and thus provide insight into the cognitive biases that individuals begin their learning with. For example, representations that develop early in life are likely to reflect fundamental biases that structure cognition. So studies with infants can reveal conceptual knowledge about event roles that is in place before children have fully learned the grammar of any particular language. Studies of newly emerging sign languages also reveal cognitive predispositions regarding concepts that lend themselves to be readily encoded in language, but in this case in the context of language being created rather than learned. Finally, child language learning can reveal whether particular role categories are more salient than others. To the extent that children's language development is not a straightforward mirror of the language input they are receiving, child language reflects “the conceptual similarities perceived by children among paradigms or structures, even where these similarities are obscured by the conventional forms of the language” (Clark & Carpenter, 1989: p. 22).

In previous discussions of how thematic roles structure cognition, roles have generally been considered in the aggregate: that is, the question “are thematic roles universal?” has been posed for thematic roles as a class. In this paper, to the contrary, we suggest the answer to this question may differ depending on the particular role considered. For this reason, we review three types of roles, addressing whether there is evidence for abstract categorization, as well as evidence for universal cognitive biases. We begin in the next section with the Agent/Patient roles, which have been by far the most extensively researched, and which provide the strongest evidence both for abstract role categories and universal biases that structure these categories. We find strong evidence for a universal bias to distinguish agents and patients. In the following section, we discuss events of transfer and crossing space, which are typically analyzed as involving a Goal/Recipient and Source. Here we address the question of whether there is a universal bias for goals to be categorized more robustly than recipients, an asymmetry that would support the hypothesized primacy of spatial cognition. We find minimal evidence for such a bias, raising the possibility that categorization of Goals and Recipients is more variable than categorization of Agents and Patients. We discuss instrumental events in the fourth section, which are typically analyzed as an Agent acting on an Instrument in order to affect a Patient. We ask whether there is a universal bias to construct an instrument category around the notion of a tool, a physical object wielded by an agent in order to achieve an outcome. Across the three types of roles considered here, we find the weakest evidence that universal biases shape role categorization for instrumental events.

In addition to questions of category abstraction and universal biases, a third crucial question for theories of thematic roles is how these categories are structured. A commonly adopted answer to the question of category representation is that roles have prototype structure (Ackerman & Moore, 2001; Dowty, 1991; Gärdenfors, 2014; Hopper & Thompson, 1980; Lakoff & Johnson, 1980; Luraghi, 2003; Primus, 1999). In Dowty’s (1991) theory of English argument realization, for example, the properties of being sentient, causative and volitional are Proto-Agent properties, whereas being causally affected and undergoing a change of state are Proto-Patient properties. Just as with the classic work on categorization conducted by Rosch and colleagues (see Hampton, 1995; Murphy, 2002; Rosch, 1975; Rosch & Mervis, 1975; Rosch, 1978), representing thematic roles in terms of prototypes provides a solution to the problem that they cannot be defined in terms of necessary and sufficient conditions. The experimental work critical to prototype theories of objects has largely not been conducted with thematic roles, for the simple reason that word meanings provide only an indirect cue to the structure of role categories. In the fifth section, we discuss how this gap could be filled in the future.

In the fifth section, we also provide a roadmap of the studies that should be conducted to fully test the nativist view of thematic roles. We argue in this paper that for multiple roles, there is only weak evidence for the nativist view. We stress, however, that the literature is more often characterized by absence of evidence (the critical studies have not been conducted) rather than negative evidence (findings contrary to the nativist view). We conclude this paper by discussing what some of these critical studies would be.

Across the cognitive sciences, thematic roles are most often invoked within the language sciences, and within linguistics, thematic roles are typically understood to be linguistic representations whose theoretical function is to explain and predict syntactic behavior, for example, through syntactically-oriented theta-roles (Baker, 1988; Chomsky, 1981; Marantz, 1984; Reinhart & Siloni, 2005), event representations (Croft, 2012; Jackendoff, 1990; Rappaport-Hovav & Levin, 1998), or logical structures (van Valin & LaPolla, 1997). At the same time, thematic roles are also event participant categories, which although reflected in language through specific linguistic structures, are not exclusively represented at a linguistic level. It therefore follows that evidence from sources outside of the confines of linguistics – including studies of infant and adult event cognition – are pertinent to understanding the full nature of thematic roles. Logically it is possible, of course, that there are multiple domain-specific thematic role representations, one that modulates syntactic behavior and another that modulates conceptual event representation, for example. In this review, we assume as a null hypothesis that, even if there are such domain-specific roles, there is also a domain-general system of event participant categories, and both linguistic and non-linguistic behavioral evidence is relevant toward understanding the nature of these categories. Finally, although we describe role abstractions as categories, we do not presuppose a particular theory of the syntax~semantics interface: the idea that there are abstract roles is compatible with theories where individual roles are representational primitives, as well as with theories in which abstract event structures such as [X CAUSE [Z HAVE Y]] map to syntax, and the categories correspond to positions in these abstract structures (Jackendoff, 1990; Rappaport-Hovav & Levin, 1998).

## Agents and patients

### Behavioral evidence for abstract agent and patient categories

Many theorists have argued that our action representations center around two maximally distinct participants (Gärdenfors, 2014; Hopper & Thompson, 1980; Lakoff & Johnson, 1980). Gärdenfors (2014), for example, proposes that “the agent and the patient of an event model are the two most central examples of thematic roles” (p. 165), and that the prototypical event involves an agent initiating a force vector that causes a change of state in a patient. The existence of abstract Agent and Patient roles in specific languages/cultures is supported by studies of event cognition and sentence comprehension. For example, adults rapidly extract agent and patient role information from visual presentation of events. Hafri, Papafragou, and Trueswell (2013) presented English-speaking adults with scenes of transitive events, such as a girl pushing a boy, for brief intervals (either 37 or 73 ms). Even at the shortest interval, participants were able to answer questions with above-chance levels of accuracy for questions such as “is the boy performing the action?” or “is the boy being acted upon?”. Although participants could succeed at this task if they only extracted event-specific roles (i.e., someone who pushes, someone being pushed), the rapid processing displayed by participants suggests they are using a more abstract schema for decoding events. The findings of Hafri, Trueswell, and Strickland (2018) support this interpretation. Participants viewed simple transitive events and performed a task unrelated to roles (they had to spatially locate a particular gender or shirt color of the people in the event). Nonetheless, participants were slower when the role (Agent vs. Patient) of the target (e.g., the person in the blue shirt) was different from the role of the target in the previous trial. This indicates that people extract role categories (specifically Agent and Patient categories) during visual event apprehension, even when they are not asked to attend to event roles.

Decades of psycholinguistic research also shows that adults activate thematic role knowledge in sentence comprehension and prediction, and most of this research focuses on the Agent and Patient roles (Altmann, 1999; Boland, Tanenhaus, Garnsey, & Carlson, 1995; Carlson & Tanenhaus, 1989; Kamide, Altmann, & Haywood, 2003; Kim & Osterhout, 2005; MacDonald, Pearlmutter, & Seidenberg, 1994; Mauner & Koenig, 2000; Trueswell, Tanenhaus, & Garnsey, 1994). For example, Mauner and Koenig (2000) found that readers could easily interpret a sentence like *the vase was sold to collect money for the charity.* As the rationale clause *to collect money for the charity* requires an agent semantically, this finding indicates that readers activated an Agent concept upon hearing the verb *sold.* By contrast, readers did experience difficulty interpreting *the vase sold to collect money for the charity*. Other studies have identified brain regions such as the left mid-superior temporal cortex that encode abstract Agent and Patient roles, during written sentence comprehension (Frankland & Greene, 2015), as well as viewing of animated videos (Wang, Cherkassky, et al., 2016). In the last decade, language comprehension research has focused on the precise timing of thematic role assignment, with debate as to whether role interpretation is delayed relative to other types of information (see Chow & Phillips, 2013; Chow, Smith, Lau, & Phillips, 2016; Kim, Oines, & Sikos, 2016; Kowalski & Huang, 2017; Kukona, Fang, Aicher, Chen, & Magnuson, 2011). Nonetheless, the idea that agent- and patient-like roles are assigned relatively rapidly during comprehension is uncontroversial.

Knowledge of abstract Agent and Patient categories is also manifest through studies of novel verb interpretation. For example, Kako (2006) asked English-speaking adults to read sentences without contentful open-class words, such as *the rom mecked the zarg.* Even for such seemingly meaningless sentences, participants rated the Subject as having more agentive properties than the Object, and the Object as having more patient-like properties than the Subject, indicating that abstract role information is linked to syntactic positions. Two-year-old children also interpret novel sentences such as *Elmo blicked Miss Piggy* as referring to an event where Elmo is an agent and Miss Piggy is a patient (Arunachalam & Waxman, 2010; Lidz, Gleitman, & Gleitman, 2003; Naigles, 1990; Noble, Roland, & Pine, 2011; Savage, Lieven, Theakston, & Tomasello, 2003) and children display sensitivity to Agent and Patient categories in sorting and language production tasks (Angiolillo & Goldin-Meadow, 1982; Braine & Wells, 1978; Braine, Brooks, Cowan, & Tamislemonda, 1993; Bridges, 1984; Corrigan & Odya-Weis, 1985; Corrigan, 1988).

This array of evidence shows that agent-like and patient-like participants are represented in terms of abstract categories. The status of putative Agent and Patient roles is not necessarily identical, however. For a range of English verbs, White, Rawlins, and Van Durme (2017) clustered arguments by semantic features considered definitive of Agent and Patient roles, such as having intention and undergoing a change of state (Dowty, 1991). While a clear Agent cluster emerged, the patient-like arguments clustered into four separate roles, subtypes of a more diffuse Patient role. The idea that Patient is a more diffuse category than Agent is consistent with findings in Hafri et al. (2013). These authors hypothesized that agents, when physically instantiated, tend to be characterized by perceptual features such as outstretched limbs and leaning forward. By contrast, patients were hypothesized to be characterized by the absence of these features. For still images of an animate agent acting on an animate patient, agents were more consistently judged as having these features than patients were judged to not have them, suggesting that the Patient role is more heterogeneous than the Agent role. Although intriguing, it is not clear to what extent this finding would generalize to other sorts of events or stimuli, such as events with inanimate agents.

### A universal bias to distinguish agents from patients

Behavioral effects from individual languages, such as priming from one type of event to another, constitute evidence for abstract event participant categories. As most of the studies reviewed in the section above were conducted with speakers of English, the question of whether the same abstract role categories are shared across the languages of the world has largely been unanswered. Nonetheless, typological evidence provides an indirect clue as to what meanings are commonly distinguished across languages, and therefore how role categories are likely to be structured. Aggregating evidence from a wide range of languages reveals a robust tendency to distinguish agents from patients. Dryer (2013) documented word orders for 1,377 languages and found that 86% of these languages distinguish agents from patients by virtue of having a dominant word order. This statistic does not imply that 14% of the sampled languages do not distinguish agents from patients; this 14% includes, for example, languages like Dutch and German, which have SVO word order in main clauses but SOV word order in embedded clauses. Siewierska (2013) sampled 380 languages and found that among those languages that use verbal person marking to code arguments, none attested a single person marker for both agents and patients, while using a different person marker for the single argument of an intransitive verb. In his study on case marking, Comrie (2013) found a similar tendency to distinguish agents from patients: across 190 languages, no language received the same case marker for agent and patient arguments of a transitive verb, but a different case marker for the single argument of an intransitive verb (see also Comrie, 1978).

This dispreference for grouping agents and patients together may not be truly universal, however: Payne (1980) documents a case-marking pattern in which agents and patients are colexified for some Iranian languages, albeit for only some pronouns. While such counter-examples suggest that distinguishing agents from patients is not a descriptive universal, these counter-examples are still compatible with a strong tendency to distinguish agents from patients. Finally, in a study of lexically-specific roles (e.g., BEATER, BEATEE), Hartmann, Haspelmath, and Cysouw (2014) studied 25 genetically diverse languages to establish whether they distinguish roles morphosyntactically. They used multidimensional scaling to analyze the similarity between each of the specific roles and found that agent-like roles (e.g., BEATER, PEELER, SIPPER) and patient-like roles (e.g., BEATEE, RECEIVED THING, COOKED FOOD) clustered distinctly from one another.

Before proceeding further, some caution is warranted. The typological evidence reviewed above – whether the same form encodes two distinct meanings – often reflects the researchers’ rough-and-ready notions of Agent and Patient. For this reason, the distinction between agents and patients that we see across diverse languages is not truly independent of the particular role categories in the minds of the researchers (see Newmeyer, 2010). Nonetheless, the fact that these rough-and-ready notions were still highly likely to be encoded by two distinct forms across languages, and that other notions do not show the same proclivity (as reviewed later), can be taken as indicative of a strong pan-human bias to draw this distinction.

While the typological evidence reveals a bias to distinguish agents from patients, evidence from emerging sign languages and infancy suggests this bias is rooted in cognition. When deaf children are born to hearing parents and are not taught a sign language, they often invent their own gestural systems (“homesign”) to communicate (Goldin-Meadow, 2003) – systems which oftentimes share the linguistic properties of established languages. Goldin-Meadow and Mylander (1998) found that homesigning children from the USA and Taiwan encoded a distinction between agents and patients in their homesign. When these children were describing events with two participants, such as a mouse eating cheese, they were more likely to produce a sign for the patient than for the agent. More importantly, agents and patients were produced using consistent word orders, for example, producing patients before acts (e.g., CHEESE-EAT). These findings show a tendency to distinguish agents from patients that emerges even among children who are not learning a linguistic system from their caregivers. Rissman and Goldin-Meadow (2017) found that a single child homesigner from the USA developed a morphological form for expressing causation, again indicating sensitivity to the category of Agent and propensity to encode this category in language (all of the causers that the child described were animate, thus agents).

Grammatical devices for distinguishing agents from patients also emerge early in sign language communities. New sign languages come into being when deaf people join a community where they are not taught an existing sign language but are free to sign with each other, as when a community opens a school for special education, or high rates of congenital deafness lead to many deaf people within extended families. Dynamics of language emergence are often studied by separating signers into distinct generations or “cohorts”: the first generation of signers has a signing community but does not receive a sign system as input. Subsequent generations learn the language that the older deaf members of the community had been using. Nicaraguan Sign Language (NSL) emerged about 50 years ago: even the earliest cohort of NSL uses word order to distinguish animate agents from inanimate patients (although word orders were more variable when both the agent and patient are animate; Flaherty, 2014). Ergin, Meir, Ilkbaşaran, Padden, and Jackendoff (2018) studied descriptions of transitive events among speakers of Central Taurus Sign Language (CTSL), which emerged about 50 years ago. The first generation of CTSL did not appear to use a consistent linguistic device to distinguish agents from patients, but the second and third generations did, using word order, spatial reference, character assignment, and causal chaining. The village sign language Al-Sayyid Bedouin Sign Language (ABSL) emerged about 80 years ago – when signers from the second generation of ABSL produced utterances with an agent and a patient, they consistently used SOV word order (Sandler, Meir, Padden, & Aronoff, 2005; Padden, Meir, Sandler, & Aronoff, 2009). Together, these results demonstrate that when signers in different cultures around the world create new languages, distinguishing agents from patients has high priority.

Experiments with infants provide a final piece of evidence for a cognitive bias to distinguish agents from patients. Before infants have learned verbs, adpositions, or syntactic structures of any particular language, they represent events in terms of conceptual structures thought to be definitive for agent- and patienthood (see Carey, 2009; Csibra & Gergely, 2007; Kelso, 2016 for review). For example, 6-month-old children represent causal relations between objects (Leslie, 1984a; Leslie & Keeble, 1987; Meltzoff, Waismeyer, & Gopnik, 2012; Muentener & Carey, 2010; Saxe, Tenenbaum, & Carey, 2005; Saxe, Tzelnic & Carey, 2007). Infants also represent actions in terms of the goals of an agent, an important precursor for understanding intentionality (Adam, Reitenbach, & Elsner, 2017; Csibra, Biro, Koos, & Gergely, 2003; Csibra, Gergely, Koos, & Brockbank, 1999; Gergely & Csibra, 2003; Krogh-Jespersen & Woodward, 2014; Leslie, 1984b; Wagner & Carey, 2005; Woodward, 1998; Woodward, 2003). For example, Woodward (1998) showed that by 6 months of age, infants analyze reaching events in terms of the goals of the agent (e.g., the object the agent is reaching for), rather than a perceptually organized analysis of the agent’s movements. Infants are also sensitive to social aspects of an agent’s action, preferring pro-social, helping agents to antisocial, hindering agents (Hamlin, Wynn, & Bloom, 2007; Hamlin, Wynn, Bloom, & Mahajan, 2011). Crucially, many of these experiments feature events that would be unfamiliar to infants, such as blocks and balls moving in a seemingly autonomous manner. This suggests that infants have an abstract agency schema, allowing them to interpret novel, unfamiliar events as goal-directed.

### Summary

Even if the roles Agent and Patient cannot be defined in terms of necessary and sufficient conditions, the evidence reviewed above suggests a universal bias to encode Agent and Patient categories distinctly from each other. These roles guide cognitive and linguistic processing in adults, they are distinctly coded cross-linguistically, they shape language emergence at an early stage, and are part of young infants’ conceptual knowledge. In the first section, we sketched a nativist view of thematic roles: they are cross-culturally universal, present early in life, and change little over the course of development. The strength of the evidence presented here suggests that this strong view may be true for Agent and Patient roles. It would be premature, however, to endorse this position, as too little is yet known about the structure of these roles in individual languages. Recall, for example, the research indicating the Patient role in English is more diffuse and heterogeneous than the Agent role. This may be indicative of a broader cross-linguistic trend, where a universal bias to distinguish agents from patients is in fact a universal bias to distinguish agents from all other types of participants. Alternatively, other languages may differ from English in having a more tightly clustered Patient role or a more heterogeneous Agent role. More in-depth cross-linguistic research is needed to address these possibilities, and their implications for the nativist view of thematic roles, as we discuss in the fifth section.

The strength of the findings on agents and patients have led some to the more general conclusion that thematic roles as a class are part of core knowledge (see Strickland, 2016). The range of events that we are able to represent and describe, however, is much more varied than the prototypical case of an agent acting on a patient, as demonstrated by example (1). We next consider events involving goals, recipients, and sources, and ask whether our representations of these events are shaped by universal cognitive biases.

## Recipients, goals, and sources

### Behavioral evidence for abstract recipient and goal categories

Across proposed lists of thematic roles, frequently listed candidates are Recipients (e.g., Paul gave the focaccia to Mary) and Goals (e.g., Mary walked to the bakery). Studies of adult sentence processing have shown that adults activate information about recipients and goals upon hearing verbs that encode these participants (e.g., *teach* activates recipient, *enter* activates goal) (Andreu, Sanz-Torrent, & Rodríguez-Ferreiro, 2016; Boland, 2005). More importantly, priming studies with adults show the order of thematic roles can be primed independently of syntactic structure, indicating abstract knowledge of a Goal category (Chang, Bock, & Goldberg, 2003; Ziegler & Snedeker, 2018) and a Recipient category (Cai, Pickering, & Branigan, 2012; Cho-Reyes, Mack, & Thompson, 2016; Hare & Goldberg, 1999; Köhne, Pickering, & Branigan, 2014; Pappert & Pechmann, 2014; Salamoura & Williams, 2007; Ziegler, Snedeker, & Wittenberg, 2018), categories that transcend verb-specific knowledge. For example, Chang et al. (2003) found that English speakers were more likely to produce sentences where the goal was mentioned in the second position after the verb (e.g., *the farmer heaped straw onto the wagon*) when primed by a sentence with the same thematic order (e.g., *the maid rubbed polished onto the table*) than a prime sentence where the goal was in the first position (e.g., *the maid rubbed the table with polish*).

Infants, even as young as 10 months, represent an abstract Goal category when viewing motion events. For example, Lakusta, Spinelli, and Garcia (2017a) familiarized 10- and 14.5-month-olds to events of an agentive entity moving to different goals, for example, a duck moving up to a tree (an AT-path event) or onto a box (an ON-path event). At test, infants looked longer at an event of a duck moving out of a bowl (a source path) than moving into a bowl (an IN-path event), indicating the infants had generalized IN-path events as being part of the same category as AT-path and ON-path events. Synthesizing the adult and infant studies, we find robust evidence that for motion and transfer events, people represent event participants in terms of abstract Goal and Recipient categories.

A thornier question concerns the relationship between the Goal and Recipient categories. These two thematic roles occupy a similar semantic space: in *Reba threw the ball to Ronnie*, for example, Ronnie could be construed as either a Goal or a Recipient. Nonetheless, Recipients are typically characterized in terms of transfer (of an object, or of information) to an animate participant, whereas Goals are typically characterized in terms of the endpoint of a spatial path. Are Goals and Recipients distinct categories that happen to overlap, or are they subtypes within a single overarching category? In many dominant theories of the language~cognition interface, spatial representations are considered fundamental, with non-spatial meanings extended metaphorically from spatial meanings (Gruber, 1965; Jackendoff, 1972, 1983; Heine, Claudi, & Hünnemeyer, 1991; Tyler & Evans, 2003). We might therefore expect that Goal constitutes the central member of this overarching category, with Recipient a less prototypical instance of this category – that transfer is a metaphorical extension of motion along a path.

Ziegler and Snedeker (2018) tested these two hypotheses about the relationship between goals and recipients. In a language production study with English-speaking adults, they found the order in which a goal was mentioned in a sentence did not prime the order in which a recipient was mentioned, or vice versa (e.g., hearing *the boy sprayed water on the plant*, where the goal is second, did not lead to more sentences such as *the woman fed the strawberry to the goose*, where the recipient is second). Minimally, these results indicate that in English, priming draws on distinct Goal and Recipient categories; but the results are also consistent with the stronger hypothesis that for adult English speakers, Goal and Recipient are representationally distinct. Along these lines, de Cuypere (2013) analyzed the English preposition *to* and found evidence against the proposal that *to* has a core spatial meaning: the diachronic record of *to* shows that the earliest uses of *to* were not restricted to spatial meanings.

### No universal bias to represent recipients in terms of goals

Existing behavioral and corpus studies of English do not provide support for the proposal that adults represent goals and recipients in terms of a single abstract category, with Recipient being a metaphorical extension of Goal. It is possible, however, that there is a general bias to represent recipients in terms of goals, even if this asymmetry is not manifest in English. The typological evidence does not support this possibility. Both goal and recipient markers (also called allatives and datives, respectively) have wide semantic extension cross-linguistically (Blansitt, 1988; Haspelmath, 2003; Heine, 1990; Svorou, 1994; Næss, 2008; Lambert, 2010; Malchukov, Haspelmath, & Comrie, 2010; Newman, 1996; Rice & Kabata, 2007; Wälchli & Zúñiga, 2006). For example, Rice and Kabata (2007) investigated the range of meanings expressed by allative markers across 44 languages (where the core use of an allative was defined as the goal of a motion event, as in *Jane walkedto the store*). Allative markers encode many senses beyond that of a spatial goal: in Japanese, for example, the allative marker *ni* is extended to 20 different senses (see Kabata 2000). Some extensions are more common than others cross-linguistically. Beyond marking goals, the allative extends most commonly to recipients (e.g., *Jane gave a bagel to her dog*), concepts (e.g., *the idea occurred to me*), purposes (e.g., *I left hometo join the circus*), and locations (e.g., Japanese *Musume waTokyo niiru*, “my daughter is in Tokyo”). Similarly, dative markers regularly extend beyond recipients to goals, beneficiaries (e.g., *Jane boughtmea sandwich*), predicative possessors (e.g., French: *Ce chien està moi* ‘This dog is mine (lit. to me)’), experiencers (e.g., *the proposal was outrageousto me*), malefactive sources (e.g., *I robbed him of money*), and patients (Haspelmath, 2003; Malchukov et al., 2010).

Researchers have used this sort of data (i.e., how often individual languages code distinct meanings with the same form) to construct semantic maps, in which senses that tend to be marked in the same way across languages are plotted close to each other in a visualization of the overall data (Haspelmath, 2003; Malchukov et al., 2010; Rice & Kabata, 2007). Across the board, goals and recipients are positioned close to each other on these semantic maps, although the central node of the map varies depending on whether the focus is goal or recipient extension. Other studies also do not support a strong distinction between these categories. For example, Bickel, Zakharko, Bierkandt, and Witzlack-Makarevich (2014) analyzed a genetically diverse sample of 114 languages using cluster analysis, asking whether thematic role categories could explain common patterns of non-default case marking. Their analysis revealed no evidence for a distinct cluster corresponding to the goal of spatial transfer: the cluster containing the goal argument for ‘throw,’ ‘bring,’ and ‘send’ also contained ‘give’ and ‘show.’ Similarly, in their study of 25 diverse languages, Hartmann et al. (2014) found no clusters specific to goals or recipients: ‘giving recipient’ was similar to ‘teachee,’ but also to ‘climbing goal,’ ‘wiped material,’ and ‘thought content.’ So the typological literature shows strong linkages between goals and recipients, but no evidence that one category is more robust than the other.

Turning to studies of homesign, there is evidence that child homesigners encode a Recipient/Goal category. Goldin-Meadow and Mylander (1984) studied ten homesigning children from the USA, analyzing the order in which children produced signs for agents, patients and recipients/goals (these authors’ coding collapsed recipients and goals into one category). For nine out of ten children, two word-order patterns were found: recipients were more likely to be ordered after agents, and they were also more likely to be ordered after patients. For three children, these patterns were statistically significant within the individual child’s data. Moreover, the broad range of lexical items used in the recipient/goal role suggested that these word order patterns were not the result of each child repeating a low-scope construction, such as “GIVE FOOD ME.” These results add to the literature reviewed above, and suggest people represent roles in terms of abstract categories. In addition, they show the cognitive robustness of these categories, as even child homesigners encode them through patterns of word order. In a later study, Zheng and Goldin-Meadow (2002) distinguished recipients from goals in their investigation of homesigners, and found both American and Chinese homesigning children were more likely to produce gestures for goals than recipients. This suggests goals are cognitively more central than recipients, as predicted by the idea that spatial representations are the foundation for action representation (Gruber, 1965; Heine et al., 1991; Jackendoff, 1972, 1983; Tyler & Evans, 2003)

Several studies of emerging sign languages have documented how signers describe events of transfer (for CTSL: Ergin et al., 2018, ABSL: Sandler et al., 2005; NSL: Senghas, Coppola, Newport, & Supalla, 1997). Nonetheless, we are not aware of studies that have tested whether grammatical devices for encoding goals emerge before grammatical devices for encoding recipients (or vice versa).

If there is a general bias for Goal to be a more robust category than Recipient, we would predict that sensitivity to goals emerges in infancy before sensitivity to recipients. Evidence from infants does not support this: sensitivity to goals and recipients develops around the same time (roughly the first birthday). As described above, Lakusta, Spinelli et al. (2017) found that 10-month-olds represented an abstract category of Goal. In a study of recipient encoding, Schöppner, Sodian, and Pauen (2006) habituated infants to scenes of a human-like puppet giving a flower to another human-like puppet. Infants subsequently dishabituated to a switch in the roles of the puppets (who was giver and who was recipient), but not to a switch in the spatial locations of the puppets. This asymmetry held for 10.5- and 12-month-olds, but not for 9-month-olds. Tatone, Geraci, and Csibra (2015) familiarized 12-month-olds to giving and taking events, and found infants distinguished these two events and linked the ‘giver’ and ‘taker’ roles to individual agents in the events.

Considering the close relationship we observe between goals and recipients across a range of paradigms, a first possibility is that humans are biased to represent goals and recipients in terms of a single thematic role centered around an animate goal, as in *Reba threw the ball toRonnie.* If so, this super-category would have a vast extension, so as to capture the range of meanings commonly encoded by allative and dative markers cross-linguistically: from purposes and locations to experiencers, possessors, and patients. One problem with such an account is that it may be too inclusive to have any explanatory power.

We propose a second possibility here: our representations of goals and recipients rely on two fundamental, but distinct cognitive systems – spatial cognition on the one hand and social relationships between animates on the other. The ability to represent spatial relationships is a core cognitive ability, and spatially based metaphors and abstractions are widespread in language (Lakoff, 1987). Nonetheless, social cognition is also fundamental, and the dative may best be understood as involving a relationship between animate individuals where there is some intermediary between the individuals, as in *Marnie taught JohnSpanish*. In her discussion of dative extensions cross-linguistically, Næss (2008: p. 4) points to the animacy of the recipient as a crucial factor: recipients are often affected by an event, and are therefore patient-like, but recipients are “less prototypical patients by virtue of their sentience” (see also Grimm, 2011). This perspective helps explain why some of the most common recipient-encoding verbs cross-linguistically, such as ‘show,’ ‘teach,’ and ‘ask,’ only involve transfer in an abstract sense (Haspelmath, 2011). Events of entities crossing space and events of two animates interacting often overlap, as in *Reba threw the ball toRonnie*. An animate participant may be the endpoint of a spatial path, and an animate participant may become affected by virtue of receiving an object that has crossed space. Under this second possibility, we observe categories of Goal and Recipient because there are universal biases to represent events in terms of movement along a spatial path, as well as social interaction between animates.

The evidence on goals and recipients is compatible with the nativist view of thematic roles – they may be distinct, universal categories that are present early in life and change little over development. An alternate possibility is more likely, however, given the diverse array of languages in which goals and recipients are colexified. The high semantic overlap of these roles may lead to more cross-linguistic diversity. There may be languages like English, where sentences with goals and recipients do not prime each other (Ziegler & Snedeker, 2018), but there may also be other languages where the roles *do* prime each other. As we reviewed above, there is evidence that Goal and Recipient categories are present in infancy. Presuming that infants across cultures have similar conceptual knowledge, it may be that categorization of goals and recipients shifts over the course of development, with adults categorizing these roles in more diverse ways than infants.

### Recipient/goals versus sources

The evidence reviewed above suggests the category of Goal is not more cognitively robust than Recipient. Such an asymmetry is present, however, between Goals and Sources, as in *Tyrell got a book fromthe library*. Sources are represented in terms of an abstract category. Lakusta, Spinelli et al. (2017) found, for example, that 14.5-month old infants generalized over events with sources (e.g., a plane flying out of a bowl, a bird walking away from a tree) when the source objects were highly visually salient. In addition, Lakusta and Landau (2005) found adults described source paths more often when they were primed with a source-encoding verb such as *unhook.* Nonetheless, behavioral experiments indicate the representation of sources is less robust than goals (Lakusta & Landau, 2005; Lakusta & Landau, 2012; Levine, Hirsh-Pasek, Pace, & Golinkoff, 2017; Papafragou, 2010; Regier & Zheng, 2007). For example, speakers across a range of languages are less likely to mention sources than goals when describing events (Lakusta & Landau, 2005; Lakusta & Landau, 2012; Narasimhan, Kopecka, Bowerman, Gullberg, & Majid, 2012; Papafragou, 2010), and adults show worse discrimination and memory for sources than for goals (Lakusta & Landau, 2012; Papafragou, 2010; Regier & Zheng, 2007).

This asymmetry is echoed by the typological literature. Cross-linguistically, sources are often distinguished from goals/recipients, both in the motion domain (e.g., *I walkedtothe store* vs. *I walkedfromthe store*) and the transfer domain (e.g., *I sent a lettertomy mother* vs. *I got a letterfrommy mother*) (Creissels, 2006; Rice & Kabata, 2007; Nikitina, 2009; Kabata, 2013; Bickel et al., 2014; cf. Wälchli & Zúñiga, 2006). Rice and Kabata (2007) sampled 44 genetically diverse languages and found the same marker was used for both goals and sources in only 11% of instances. Bickel et al. (2014) provide further support for this distinction: in their analysis of non-default case marking in 114 languages, they found source arguments (e.g., ‘buy-from’ and ‘get-from’) clustered distinctly from a range of other roles. Most importantly, Kabata (2013) surveyed 24 languages and found sources had a narrower scope of semantic extension than goals, consistent with the behavioral evidence showing Source is a less robust category.

Evidence from emerging sign languages and infancy supports these findings. Zheng and Goldin-Meadow (2002) studied descriptions of motion events from homesigning children in the USA and Taiwan, and found children from both groups produced gestures for goals and sources; but gestures for goals were about five times as common as gestures for sources. In addition, infants encode goals of motion events more robustly than sources. Lakusta, Wagner, O'Hearn, and Landau (2007), and Lakusta and Carey (2015) found that when 12-month-olds were familiarized to events of a bird flying from one of two sources to one of two goals, they increased their looking when the bird flew from the same source to a different goal, but not when the bird flew from a different source to the same goal (see also Lakusta, Spinelli et al., 2017). Tatone et al. (2015) also found infants encoded the absence of a recipient from a giving event more strongly than the absence of a source from a taking event.

Taken together, the typological evidence and studies with adults, homesigners, and infants provide strong evidence for a universal cognitive bias such that goals are represented more robustly than sources. A possible factor contributing to this asymmetry is that goals may provide new discourse information more often than sources do – that is, an event with a goal must also have a source, but an event with a source need not have a goal (Lakusta & Landau, 2012). The consensus that Sources are less robust than Goals, however, is potentially inconsistent with Clark and Carpenter's (1989) study of children learning English. The authors found a tendency for children to overgeneralize the preposition *from* to agents, causes, and possessors, as in *he isn't going to get hurt from those bad guys,* suggesting conceptual relatedness between sources and these three role types (see also Lakusta, Thothathiri, Mendez, & Marinkovic, 2017). Clark and Carpenter interpret this result as indicating the primacy of spatial cognition, and conclude there is a broad category of Source that includes Agent. This interpretation is difficult to reconcile with the infant research that shows infants encode agents roughly six months before they encode sources. An alternative possibility is that English-learning children overgeneralize *from* because they are uncertain about the category of meanings *from* picks out, rather than because they represent a primary Source category.

### Summary

The psycholinguistic literature provides robust evidence that (English-speaking) adults represent events in terms of Goal and Recipient categories that abstract beyond the verb-specific and event-specific level. These categories appear to be an important part of our conceptual and semantic repertoire: infants less than a year of age represent these (or similar) notions, and deaf children lacking exposure to a language model produce signs for these entities. In addition, humans robustly distinguish sources from goals, as shown through cross-linguistic research, adult psycholinguistic experiments, and studies with infants. Nonetheless, it is unclear how our concepts about recipients and goals are structured: are these subtypes of the same category, or are they distinct categories drawing on two distinct cognitive systems? Although the nativist view of thematic roles is not decisively falsified by the available evidence on recipients and goals, this evidence suggests that there is greater variability in how these event participants are categorized than in how agents are categorized.

## Instruments

### Evidence for an instrument category?

We now turn to the final type of thematic role reviewed here – Instrument, introduced in (1) – and explore the evidence for an Instrument category. Instrument has a long-standing place in discussions of thematic roles, dating back at least to the Sanskrit grammarian Pāṇini. Fillmore (1968) proposed that Instrument is one of a small set of universal roles. Instruments are often linked to concepts of agency and causation, as the extension of an agent or standing in a part-whole metonymic relation (DeLancey, 1991; Dowty, 1991; Grimm, 2007; Luján, 2010; Rissman, Rawlins, & Landau, 2015; Rissman & Rawlins, 2017; Schlesinger, 1989; van Valin & Wilkins, 1996). That is, the function of an instrument is thought to be essential to its meaning: being *used* by an agent to achieve something. Like other thematic roles, Instrument has been described as having prototype structure: Luraghi (2001: 388) characterizes a prototypical instrument as “an inanimate manipulable entity which occurs in a controlled state of affairs, where an agent acts intentionally.” We refer to this as the “tool” definition for convenience. This tool prototype is exemplified by (1a), *Janine ate the custard witha spoon*, and the other examples in (1) are assumed to be less prototypical members of the category.

There is surprisingly little empirical evidence for the Instrument category. Psycholinguistic studies have shown specific verbs can activate instrumental concepts (Andreu et al., 2016; Koenig, Mauner, & Bienvenue, 2002; Koenig, Mauner, & Bienvenue, 2003; Rissman et al., 2015). For example, in Koenig et al. (2003), participants read sentences such as *which sword did the rebels behead the traitor king with*? When the verb encoded the presence of an instrument (e.g., *behead* as opposed to *kill*), participants were faster at recognizing the *wh*-filler (e.g., *sword*) was an instrument rather than a patient. This result indicates verb-specific encoding of instruments rather than an abstract Instrument category. The two words in English that most commonly introduce instruments are *with* and *use.* These words do not obviously encode Instrumental categories, however (Rissman & Rawlins, 2017): consider *she impressed the committee withher confidence* and *she usedthe hour before lunchto write a letter.* Rissman and Rawlins (2017) found the only generalization uniting this range of meanings was: an Instrument is “an entity, either concrete or abstract, acted on by an agent as part of a larger event.” In the linguistics literature, a commonly-used diagnostic to identify an Instrument is if both the *with*- and *use*- versions of the sentence are possible (e.g., *he ate ice cream witha spoon*; *he useda spoonto eat ice cream;* see Koenig et al., 2008; Lakoff, 1968; Nilsen 1973). Problematically, this diagnostic assumes *a priori* an Instrument category centered around the tool prototype.

### Universal biases

The existence of an Instrument category is under-supported empirically. Nevertheless, there could be a universal bias to construct a participant category around the concept of a tool. As discussed above, instruments are often analyzed as extensions of agents, and this possibility is supported cross-linguistically: agent/instrument colexification is common outside of the Indo-European language family (Stolz, 2001). In the semantic map constructed by Narrog and Ito (2007) based on 200 languages, the roles agent, passive agent, ergative, and cause/reason are in close proximity to the instrument role. Children learning English also appear to be sensitive to the relationship between agents and instruments. Braine and Wells (1978) trained 5-year-olds to place Actor, Object, and Instrument tokens on pictures such as a soldier shooting with a gun (Actor + Instrument tokens). In generalization trials with three participants, such as *the bear used a bat to break the clock*, children’s placement of the three tokens was highly accurate. But in trials with only two participants, such as *the cake was cut with a knife*, children were uncertain as to whether the non-object (here, the knife) was an Actor or an Instrument.

In addition to the agent-instrument linkage, typological research shows that instruments are closely linked to comitatives (sometimes labeled “companions,” as in *Coco went to the storewith Joelle*) (Heine et al. 1991; Lakoff & Johnson, 1980; Luraghi, 2001; Nilsen, 1973; Schlesinger, 1995). Narrog and Ito (2007) found that having a single form for instruments and comitatives was one of the most frequent colexifications in their data set. The behavior of English *with* lead Lakoff and Johnson (1980) to propose a cross-culturally universal metaphor in which an instrument is conceptualized as a companion. Contrary to this, it appears the comitative/instrumental colexification is overwhelmingly common only in Indo-European (Stolz, 1996, 2001), and not the rule when considering a diverse range of language families.

In addition to agents and comitatives, instrumental meanings colexify with themes, as in *Gabe filled the glasswith orange juice*. In their sample of 114 languages, Bickel et al. (2014) did not find a clear separation between themes of spatial transfer (such as for *put* and *throw*) and instruments (such as for *cover* and *hit*). In other words, the object being thrown (e.g., a ball) is often marked the same way as the object being used to cover (e.g., a blanket). These ‘throwee’ and ‘cover’ microroles demonstrate a linkage between themes and instruments: both move through space from one location to another, and both can be used intentionally to affect another participant. Locative/instrumental colexification is also common: for example, in *Troy cut the breadby hand* and *Troy went to Amsterdamby train*, the train is both a means of travel and a container (see Luján & Ruiz Abad, 2014; Luraghi, 2004; Narrog & Ito, 2007).

In sum, the typological literature exhibits a swathe of semantic relationships between instruments and other meanings. Given this, it is not obvious how one could capture all cross-linguistically attested patterns within a single semantic notion. The typological data are consistent with the proposal that there are no universal biases shaping how we construct instrumental categories. On the other hand, the typological data could also be consistent with the proposal that humans are biased to represent instrumental events in terms of a tool prototype, a possibility we consider below.

The idea that tools are cognitively central is supported by work in the infancy literature. By their first birthday, infants represent tools as means through which an agent achieves her goals (Biro & Leslie, 2007; Hofer, Hauf & Aschersleben, 2005; Jovanovic et al., 2007; Sommerville & Woodward, 2005). Stavans and Baillargeon (2018) found that 4-month-old infants were able to individuate objects when they had seen those objects used as tools, suggesting infants use information about tool use to assist individuation. Crucially, infants did not individuate objects when they were not used in a typical tool-like fashion (e.g., when the experimenter squeezed a pair of tongs together above a toy, rather than using the tongs to lift a toy). When 11- to 12-month-old infants observe novel objects being used functionally (i.e., as tools), they categorize the objects based on the part of the object that affords the function, rather than overall similarity (Träuble & Pauen, 2007). Moreover, 13.5-month-olds are able to learn an association between a tool and an end-state, even when the means relationship between the two is opaque (e.g., after covering a banana with a flowerpot, the banana magically becomes peeled; Hernik & Csibra, 2015). The evidence that infants can represent the functions of tools, and that they use this to help them individuate objects, suggests tools are a cognitively robust category. In addition, although English *with* is highly polysemous, the instrumental sense of *with* is one of the earliest senses acquired by children (Clark & Carpenter, 1989; Tomasello, 1987).

Relatively few studies have addressed how instrumental meanings are encoded in emerging sign languages, although we do know that homesigning children label tools: Rissman, Horton, and Goldin-Meadow (2018) studied nine children from Guatemala, Nicaragua, the USA, and Taiwan (age range: 2;11 – 12;0), and found all children produced signs referring to tools. This evidence does not, however, reveal the *categories* that these homesigners used to represent tools. Several studies have investigated the morphological structure of instrumental signs in ABSL and CTSL (Hwang, Tomita, Morgan, Ergin, et al., 2016; Padden, Meir, Hwang, Lepic, Seegers, & Sampson, 2013), although these studies have not focused on the relationship between instruments and related meanings (e.g., agents, comitatives, themes), so this still requires further investigation.

To summarize, the nativist view of thematic roles may be correct for Instruments, such that the tool is the prototype within the Instrument category, and it could be a category with universal relevance. However, the available evidence about instruments is more limited than for either agent/patient roles or recipient/goal/source roles. Although the current evidence does not contradict the nativist hypothesis, there is little linguistic or behavioral evidence that directly supports it either. Instead, we see evidence for linkages between instruments and a range of other roles, from agents and comitatives to locatives and themes.

### The future of research on thematic roles

In many ways, the current literature provides a detailed view of event participant categories and how they influence linguistic and cognitive behavior across a range of human populations. But there are a variety of gaps in the literature that prevent us from knowing whether the nativist view of thematic roles is correct for even a single role. In this section, we discuss how these gaps could be filled. One of the most notable omissions concerns the structure of the categories. In the section below, we discuss the prominent proposal that thematic roles are structured in terms of prototypes, and some of the limitations in extending prototype theory to thematic roles. We also provide a roadmap for the types of studies that should be done to fully test the nativist view of thematic roles.

### Thematic roles and prototypes

There is already ample evidence that thematic roles defined in terms of necessary and sufficient conditions are not universal, at least not as far as the syntax~semantics interface is concerned. For example, both nominative and ergative case markers introduce agents, but these markers have different extensions: only nominative case marks the single argument of an intransitive verb such as *run*. Analyzing thematic roles in terms of prototypes is the most common response to the observation that roles are not easily defined in terms of necessary and sufficient conditions. And, given extensive cross-linguistic variability, the proposal that thematic roles are part of core knowledge may depend on thematic roles having prototype structure.

The evidence reviewed illustrates that roles are represented in terms of abstract categories and are present in infants and deaf signers creating new languages. But the literature reveals little about whether these categories have prototype structure, and whether the prototypes are the same across languages. Relatively few studies have used methods comparable to the classic work in categorization conducted by Rosch and colleagues (see Hampton, 1995; Rosch, 1975; Rosch & Mervis, 1975; Rosch, 1978). We summarize the relevant work here, and suggest how it could be applied to the study of thematic roles in the future.

The behavioral phenomena supporting prototype representations are well-documented (see Geeraerts, 2010; Hampton, 2006; Murphy, 2002; Rosch, 1978; for review). For example, when people judge whether item X is a member of category Y, response times are faster when X is a more prototypical member of the category (e.g., people judge that *robins are birds* more quickly than *penguins are birds*). Similarly, when people are asked to list members of a category, prototypical members are mentioned earlier, and more often (across people). Also, hedging language is more acceptable for non-prototypical members of a category than prototypical members (*a penguin is technically a bird* vs. *a robin is technically a bird*).

Although the idea that thematic roles have prototypes is widespread, the classic methods used to establish prototypes have rarely been applied to the study of thematic roles, for the critical reason that these methods rely on lexical labels (e.g. *bird*). As described in the introduction, role categories often do not map cleanly onto words. For example, neither English *with* nor *use* on their own point to the category Instrument, as discussed above. Only the intersection of these terms corresponds to intuitions about what a prototypical Instrument might be. Given this asymmetry between event participant categories and lexical meanings, the same methods that support prototype theories cannot be directly extended to the study of thematic roles. As a consequence, claims about thematic role prototypes are often put forth without extensive behavioral or cross-linguistic evidence, as pointed out by Rice (1996).

Geeraerts (2010: 189) summarizes four types of prototypicality effects: (1) differences of typicality and membership, (2) membership uncertainty, (3) clustering into family resemblances, and (4) absence of necessary-and-sufficient definitions. Not all categories demonstrate all four of these effects. The category *fruit*, for example, demonstrates both the first and second effects: first, apples and pomegranates are both fruit, but an apple is a more typical exemplar than a pomegranate. Demonstrating membership uncertainty, many English speakers are unsure whether olives and coconuts are fruits. The English category *bird*, however, demonstrates the first characteristic but not the second: a robin is a better example of a bird than a penguin, but for English speakers with a particular educational background, the boundaries of the category *bird* are discrete rather than fuzzy: a bat is not a bird, a flying squirrel is not a bird.

Which of these four characteristics do thematic role categories demonstrate? As described in the first section, they cannot be convincingly defined in terms of necessary and sufficient conditions. In the linguistics literature, thematic roles are often thought to demonstrate the first effect, graded typicality among members. Most notably, Dowty (1991) invokes prototypes to explain the puzzle that in English, agents generally appear as Subject and patients as Object (*Tasha kissed the baby* has to mean that Tasha was the one doing the kissing). Nonetheless, many types of participants appear as Subject that are not particularly agentive, such as in *Tasha believed the news.* Dowty’s proposal is that for each English verb, the argument with the most Proto-Agent properties (e.g., sentience, causation, and volition) is realized as Subject, and the argument with the most Proto-Patient properties (e.g., affectedness, being an incremental theme) is realized as Object. An argument does not have to be a good example of an agent to appear in Subject position; it just has to have some Proto-Agent properties. Grimm (2011) organizes Dowty’s Proto-Role properties into a lattice structure, such that arguments with all of the agentive properties are better examples of the category Agent than arguments with fewer of these properties. This Proto-Role approach has been extended to a range of languages beyond English (Ackerman & Moore, 2001; Grimm, 2011; Primus, 1999).

This type of linguistic graded typicality effect has not, however, been demonstrated for a wider range of behavioral data. Nonetheless, existing behavioral methods can be extended to test for graded typicality of thematic roles, as event processing studies reveal asymmetries in how people identify and remember different types of participants. As described in the third section, Lakusta and Landau (2012) found in a change-detection task that goals were remembered better than sources. Dobel, Gumnior, Bölte, and Zwitserlood (2007) conducted an event identification task, where participants viewed rapidly-presented scenes of an agent transferring something to a recipient (scene duration: 100–300 ms). Although both the agent and the recipient were animate, participants were less accurate at naming the recipient than the agent in the 200- to 300-ms window. If thematic roles are structured such that some members of the category are more typical than others, then a reasonable linking hypothesis is that in such tasks, processing and memory will be more robust for the typical members than for the atypical ones, *ceteris paribus*.

Consistent with this reasoning, note that the asymmetry between source and goal demonstrated by Lakusta et al. (2007) is only observed for animate entities such as a duck. When Lakusta and Carey (2015) showed 12-month-olds events of a balloon crossing from a source to a goal, infants did not increase their looking to either the different-goal events or the different-source events. This suggests the most robustly encoded notion of Goal is narrower than the endpoint of a crossing of space – it is the destination reached by a self-directed entity. This in turn might suggest that the latter is the more prototypical instance of the Goal category than the former.

In the Recipient domain, one possibility is that physical transfer is central: cross-linguistically, verbs of giving are by far more common than other types of three-argument verbs (Newman, 1996). Along similar lines, Tatone et al. (2015: p. 48) argue that infants possess a giving action schema, and that “humans are equipped with a specialized cognitive adaptation for understanding and participating in resource exchange.” If the recipient of a physical transfer event is more prototypical than the recipient of a communication event, then memory and processing should be better for the former than for the latter. In the third section, we proposed that the Goal and Recipient categories are underpinned by two fundamental cognitive systems (spatial vs. social cognition), and that one role is not more primary than the other. We are not aware of any studies that have directly tested whether processing of goals is more robust than processing of recipients, so this remains a matter for future investigation.

The second prototypicality characteristic discussed by Geeraerts (2010) is that categories may have fuzzy boundaries, leading to uncertainty about whether a particular exemplar is a member of the category. In practice for researchers trying to delimit where one thematic role ends and another begins, this uncertainty is all too familiar. Consider, for example, the contrast between *Martha ate the custard witha spoon* and *Martha sprayed the ferns withwater*. Some researchers have analyzed the two underlined participants as members of different categories, Instrument and Locatum, respectively, under the justification that the latter sentence is fundamentally an event of a substance crossing space, rather than an event of tool manipulation (Jackendoff, 1990). Other researchers have classified both the spoon and the water as Instruments, as they are both used by an agent to achieve a goal (Koenig et al., 2008). This issue of membership uncertainty has led to the widespread confusion observed by Dowty and Newmeyer at the outset of this paper. Drawing on the current example, the water may be an *atypical* member of the category Instrument, a *typical* member of the distinct category Locatum, or it may not fit neatly into any category, as with the noun *olive*, discussed above. Priming experiments such as in Ziegler and Snedeker (2018) can address whether instruments and locatum participants are part of the same category.

The third prototypicality characteristic is family resemblance. A well-known example concerns the different senses of the English preposition *over* (e.g., *the plane flew over the city*, *he put his hands over his face*): these have been argued to be related by meaning chains, where some senses involve a vertical arrangement and others involve physical covering, etc. (Brugman, 1988; Lakoff, 1987; Taylor, 2003). These types of meaning chains are explicit in the Proto-Role proposals of Dowty (1991) and Grimm (2011), among others. The typological literature reviewed in the second to fourth sections also reveals which semantic relationships are particularly relevant for category membership across languages. Goals, for example, are closely related to locations and purposes, and instruments are closely related to agents and themes. So we have a relatively well-developed understanding of how similarity is defined within the semantic space of event participant roles. In fact, the clearest alternative to the proposal that thematic roles have prototype structure is that participant roles are semantically related through chaining, without the presence of a central reference point. In a study of how the senses of English words changed over time, Ramiro, Srinivasan, Malt, and Xu (2018) found that a model based on nearest neighbor semantic chaining was more efficient than a model based on a central prototype. Whether the same holds for thematic roles is not known.

An additional question concerns the types of semantic features that comprise the family resemblance structures in thematic roles. Dowty and Grimm, *inter alia*, propose abstract features such as intentionality and causation. In the event cognition experiments of Hafri et al. (2013), perceptual features such as leaning and having outstretched limbs influenced how quickly a figure was categorized as an agent. Verb-specific typicality information (e.g., that the agent of a frightening event is likely to be scary and unfriendly) has also been argued to be part of thematic role structure (Ferretti et al., 2001; McRae et al., 1997; see also Brown & Dell, 1987). Each of these types of features may be part of role prototypes – it is unknown, however, whether different cognitive and linguistic behaviors rely on different sets of features.

###  roadmap for future research

The previous section described methods for testing whether event participant categories have prototype structure. These methods would enable researchers to establish not only whether event participants are represented in terms of abstract categories, but also to propose analyses of how the categories are structured. In this section, we propose a variety of steps that should be taken to more decisively answer the question of whether event participant categories are influenced by universal cognitive biases and whether the nativist view of thematic roles is correct.

The majority of experimental studies on event cognition and memory have been conducted with speakers of English. Studies in other semantic domains have tested whether prototypes are variable across languages (color: Regier, Kay & Cook, 2005; containers: Malt, Sloman & Gennari, 2003). Experimental work on thematic roles should also be expanded to a broader range of languages to address both the question of role abstraction as well as prototypicality. Although Ziegler and Snedeker (2018) did not find syntactic priming between goals and recipients in English, such priming may well be observed for other languages. As described above, experimental work could also test whether thematic roles demonstrate prototype effects, such as typicality of membership and membership uncertainty. It is entirely possible that, even if all the thematic roles summarized in this paper are represented in terms of prototypes in a particular language, the prototypes are not identical across languages and cultures. Much of the typological and language emergence evidence reviewed in the second section documented that agents and patients are strongly *distinguished* across languages. However, there may be a universal bias to distinguish agents and patients, without these categories having the same prototypes and category structure in each culture. In addition, the modeling in White et al. (2017) raises the possibility that prototypes are more variable across languages for agents than for patients. If such variability is attested, it opens a range of questions that have scarcely been asked: for example, how are event participant categories molded by culture, environment, and history, and whether the particular distinctions made in our language affect our conceptual representations of thematic roles.

Universal biases can influence not only structure within a category but also relationships across categories. We reviewed a variety of evidence that some types of roles appear to be more cognitively prominent than others. As argued by Blake (1994), there is a general hierarchy concerning which cases tend to be expressed in languages with inflectional case – nominative case is most prevalent, followed by accusative and ergative case, then genitive, dative, and the others (see also Levin & Rappaport-Hovav, 2005). Role prominence is also manifest in the argument/adjunct distinction, where agents and patients are likely to be encoded in syntactically prominent argument positions, but instruments are more likely to be encoded in syntactically peripheral adjunct positions (Rissman et al., 2015; Schutze, 1995; Sedivy & Spivey-Knowlton, 1994). Asymmetries such as these are not only reflected in language. As described in the third section, infants encode sources less robustly than goals, and adults have poorer memory for sources than goals. Dobel et al. (2007) demonstrate better naming accuracy for agents than recipients (see also Wilson, Papafragou, Bunger, & Trueswell, 2011). Moreover, in his discussion of the Recipient role, Haspelmath (2011) points out that languages do not tend to have a large number of physical transfer verbs, cautioning that we cannot “say with confidence that physical transfer verbs of possession tend to be the major class” and that “we have to accept that the comparative concepts R [recipient] and T [theme] do not capture the same range of phenomena as the concepts A [agent] and P [patient], and are thus much less significant” (p. 559). Experimental work across a diverse range of languages is needed to test whether role asymmetries can be explained by universal cognitive biases.

Another crucial question for future research concerns the relationship between thematic roles as linguistic objects and thematic roles as conceptual categories. As mentioned previously, the syntax~semantics interface is variable across languages. This variability is also manifest within a single language. For example, in Rissman and Rawlins (2017), both *use*-sentences and *with*-sentences entail the presence of an agent – the truth-conditions of both *Chloe used scissors to cut the dress* and *Chloe cut the dress with scissors* contain the proposition *Ag(e) = Chloe*. Nonetheless, *use* and *with* encode different instrumental features: *use* requires an intentional agent. This variability does not, however, falsify the hypothesis that the category Agent is universal. If we allow that *Ag(e)* has prototype structure, where an unintentional agent is still an Agent, then both *use* and *with* are compatible with this category. For Agent to truly be universal, however, it must first be the case that in English, the categories demonstrated by non-linguistic event cognition experiments are the same as the categories needed to account for natural language semantics (e.g., the meanings of *use* and *with*). It must then be the case that this parallelism holds in all languages. If we find any language where this parallelism does not hold, this suggests domain-specific thematic role representations that may be structured in different ways. A category such as *Ag(e)*, for example, may need to be relativized to a particular language or linguistic construction.

## Conclusion

One of the fundamental questions of cognitive science concerns the ways in which people categorize the world around them, and the extent to which humans form similar or different categories across languages and cultures. From a domain-general perspective, the study of thematic roles is the study of event participant categories, and studying these categories provides an opportunity to gain insight into the interface between conceptual and semantic knowledge. Many psychologists working on concept representation do not distinguish between the semantic category picked out by the particular English word *dog*, and categorization of furry, four-legged domestic animals at a conceptual level. As thematic roles are largely not labeled through open class lexical items, such conflation is not possible in the domain of event participant categories.

At the outset of this review, we described two poles in theorizing about thematic roles – thematic roles are part of core knowledge or they are scholarly fictions. The evidence we have reviewed from adult psycholinguistics, development, typology, and emerging sign languages makes it untenable to maintain the view that thematic roles are fiction. At the same time, it is also premature to infer the contrary – there is simply insufficient evidence to conclude that thematic roles as a class constitute core knowledge. What the data do show is that for the best-studied roles, there is evidence for abstraction, scaffolded by universal cognitive biases. The exact nature of these abstractions – i.e., whether they have the same content and structure across languages – is not clear. In sum, despite the substantive literature reviewed herein, many foundational questions about thematic roles remain unanswered: what the structure of event participant categories is in individual languages, and whether, for lesser-studied roles such as Instrument, there are universal biases. Nonetheless, through an integration of diverse methods and sources of evidence, answers to these foundational questions are well within reach.

## Footnotes

1 In this paper, role labels such as "Instrument" are used intensionally to refer to a proposed mental category. Lower-case labels such as "instrument" are used extensionally to refer to a particular entity which may or may not be a member of a role category.
